# Molecular Pathways of Cellular Senescence and Placental Aging in Late Fetal Growth Restriction and Stillbirth

**DOI:** 10.3390/ijms22084186

**Published:** 2021-04-18

**Authors:** Anna Kajdy, Jan Modzelewski, Aneta Cymbaluk-Płoska, Ewa Kwiatkowska, Magdalena Bednarek-Jędrzejek, Dariusz Borowski, Katarzyna Stefańska, Michał Rabijewski, Andrzej Torbé, Sebastian Kwiatkowski

**Affiliations:** 1 Department of Reproductive Health, Centre of Postgraduate Medical Education, Żelazna 90 St., 01-004 Warsaw, Poland; jmodzelewski@cmkp.edu.pl (J.M.); mirab@cmkp.edu.pl (M.R.); 2 Department of Gynecological Surgery and Gynecological Oncology of Adults and Adolescents, Pomeranian Medical University, Al. Powstańców Wlkp. 72, 70-111 Szczecin, Poland; aneta.cymbaluk@gmail.com; 3 Department of Nephrology, Transplantology and Internal Medicine, Pomeranian Medical University, Al. Powstańców Wlkp. 72, 70-111 Szczecin, Poland; ewakwiat@gmail.com; 4 Department Obstetrics and Gynecology, Pomeranian Medical University, Al. Powstańców Wlkp. 72, 70-111 Szczecin, Poland; m.bednarekjedrzejek@gmail.com (M.B.-J.); torbea@wp.pl (A.T.); 5 Clinic of Fetal-Maternal, Gynecology and Neonatology, Collegium Medicum, Nicolaus Copernicus University in Bydgoszcz, Łukasiewicza 1 St., 85-821 Bydgoszcz, Poland; darekborowski@gmail.com; 6 Department of Obstetrics, Medical University of Gdańsk, Mariana Smoluchowskiego 17 St., 80-214 Gdańsk, Poland; kciach@wp.pl

**Keywords:** placental aging, cellular senescence, FGR, SGA, stillbirth, telomere homeostasis, oxidative stress, SAHF, senescence-associated secretory phenotype

## Abstract

Abnormally accelerated, premature placental senescence plays a crucial role in the genesis of pregnancy pathologies. Abnormal growth in the third trimester can present as small for gestational age fetuses or fetal growth restriction. One differs from the other by the presence of signs of placental insufficiency and the risk of stillbirth. The majority of stillbirths occur in normally grown fetuses and are classified as “unexplained”, which often leads to conclusions that they were unpreventable. The main characteristic of aging is a gradual decline in the function of cells, tissues, and organs. These changes result in the accumulation of senescent cells in mitotic tissues. These cells begin the aging process that disrupts tissues’ normal functions by affecting neighboring cells, degrading the extracellular matrix, and reducing tissues’ regeneration capacity. Different degrees of abnormal placentation result in the severity of fetal growth restriction and its sequelae, including fetal death. This review aims to present the current knowledge and identify future research directions to understand better placental aging in late fetal growth restriction and unexplained stillbirth. We hypothesized that the final diagnosis of placental insufficiency can be made only using markers of placental senescence.

## 1. Introduction

The aging of the placenta is a physiological process that occurs as pregnancy advances. Abnormally accelerated, premature placental senescence plays a crucial role in the genesis of pregnancy pathologies [1]. It has been described in obstetric complications such as abnormal fetal growth, preeclampsia, preterm birth, the premature rupture of membranes, and stillbirth. One of the greatest challenges of perinatal medicine is late fetal growth restriction and term fetal death resulting in stillbirth [2,3]. Abnormal growth in the third trimester can present as small for gestational age fetuses (SGA) or fetal growth restriction (FGR) [4]. It is believed that one differs from the other by the presence of ultrasonographic signs of placental insufficiency [5]. SGA and FGR both carry a certain degree of stillbirth risk, but most term deaths are found in normally grown fetuses [6]. A confounding relationship has been described between smallness and stillbirth. Stillborn neonates progressively lose 20–25% of their body weight in utero [7]. Most stillbirths occur in normally grown fetuses. Only 30–40% of stillbirths after 32 weeks are classified as below the 10th centile [8,9,10,11,12]. The majority of stillbirths are classified as “unexplained”, which often leads to the conclusion that they were unpreventable [11,13]. Although it is widely hypothesized that the identification of a placental pathology pre- or postnatally could be the key to understanding these deaths [8].

The main characteristic of aging is a gradual decline in the function of cells, tissues, and organs. These changes result in the accumulation of senescent cells in mitotic tissues. Senescent cells are cells that begin the aging process that disrupts tissues’ normal function by affecting neighboring cells, degrading the extracellular matrix, and reducing tissues’ regeneration capacity. The latter results from decreasing the number of stem and progenitor cells [14]. Different degrees of placental insufficiency result in the severity of fetal growth restriction and its sequelae, including fetal death.

This review aims to present the current knowledge and identify future research directions to understand better placental aging in late fetal growth restrictions and unexplained stillbirths. We hypothesize that the final diagnosis of placental insufficiency can be made only using markers of placental senescence. This could help delineate the more subtle forms of placental insufficiency that have a long-term effect on health and explain stillbirth in an otherwise healthy pregnancy.

## 2. Biology of Cellular Senescence and Aging

Every cell, tissue, and organ in the human body has a programmed lifespan. Aging is a physiological process of all living beings. It is defined as a progressive loss of the function of cells, tissues, and organs [15]. This results in a reduced ability to adapt to environmental stressors and increased morbidity and mortality [16]. Aging results from the disruption of specific molecular pathways and structures, which leads to a loss of homeostasis and homeodynamic failure [17]. Homeodynamics is a system designed to maintain the functional capacity over time. It dynamically reorganizes and resets the equilibrium in response to external and internal stressors [15]. Individuals vary significantly in the onset, progression, and extent of aging. This is reflected in the functional capacity, which is a direct measure of the cellular and organic ability to operate properly and is under the influence of both genes and the environment [15,16]. Other approaches to aging have been described. Among others, epigenetic aging has been described as a form of aging completely or semi-independently from senescence. Epigenetic clocks are used as accurate markers of age acceleration. Placental clocks have been applied in preeclampsia, sex differences in fetal growth, including SGA risk, and other pregnancy complications [18].

The progressive accumulation of senescent cells in mitotic tissues is the leading cause of aging. Biomarkers of cellular senescence can be used as markers of tissue aging. Senescent cells affect the functioning of tissues and organs. They change neighboring cells’ expected behaviors, degrade their structural components, and accelerate tissue regeneration capacity loss [14]. Many mechanisms of maintenance and theories regarding aging have been described. These include DNA damage, repair, synthesis, detection, the clearance of defective proteins, lipids, organelles, cells, defense against injury, pathogens, the elimination of free radicals, mechanisms of mitochondrial damage, and immunosenescence theories [15].

Cellular senescence can be viewed as a terminal state of cell growth. It can be triggered by a wide range of both internal and external stressors [1]. Oxidative stress is the best-described mechanism inducing cell senescence [19,20]. Free radicals cause DNA damage, which, in result, initiates apoptosis [21]. DNA damage can include telomeric or genomic DNA. Short or dysfunctional telomeres induce the apoptosis of mitotic cells [22,23]. DNA damage response mediators, overexpression of the oncogenic renin-angiotensin system (RAS), transmitting and activating signaling such as RAS-p21, mitogen-activated protein kinase (MAPK), mammalian target of rapamycin complex (mTORC), chromatin disruption, oncogene expression, mitochondrial dysfunction, and oxidative stress induced by reactive oxygen species (ROS) can also generate senescence [24,25,26,27].

**Figure 1:**
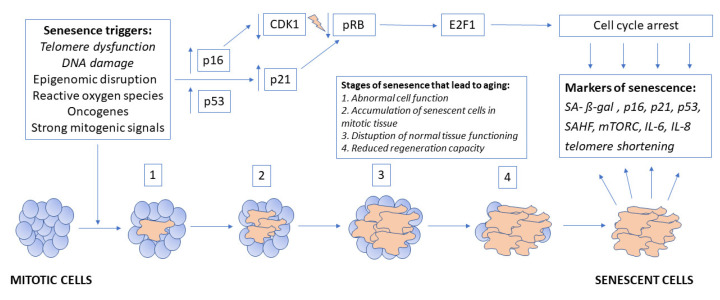
Pathophysiology of placental aging.

Senescence stressors activate one of two or both pathways that regulate this process. These pathways are controlled by tumor-suppressor protein p53, pRB, and cyclin-dependent kinases (CDKs). Activated p53 induces the expression of p21, which is a CDK inhibitor. p16 and p21 suppress the phosphorylation and inactivation of pRB. The active form of pRB suppresses a transcription factor, E2F1, which irreversibly blocks the cell cycle. This leads to the reorganization of heterochromatin, which accumulates in senescent cells in the form of senescence-associated heterochromatin foci (SAHF) [28,29,30] Figure 1.

## 3. Role of Physiological Placental Aging in Human Parturition

The trophoblast is the first cell lineage to differentiate at the stage of the blastocyst. As differentiation continuous, it follows the villous and the extravillous pathways. At implantation, the early syncytiotrophoblast is generated. It increases in size by feeding on mononucleated cytotrophoblast cells [31]. The latter cells proliferate, differentiate, and syncytially fuse with the syncytiotrophoblast to increase and maintain this multinucleated layer. During the early stages, the syncytiotrophoblast is invasive and helps to penetrate the uterine epithelium. At about day 12 post-conception, the cytotrophoblast cells start to penetrate through the syncytiotrophoblastic mass, moving toward the first branches that extend into the intervillous space, thus resulting in the formation of villous trophoblast cells. Later, the cytotrophoblast cells reach the maternal side of the syncytiotrophoblast mass [32]. This is the time of the first contact of mononucleated trophoblast cells with the maternal decidual stroma and establishment of the extravillous trophoblast [33].

Terminal villi are built from an outer layer of trophoblast that forms a grid of connective tissue and small blood vessels. Cytotrophoblast cells rise and proliferate from this villous trophoblast layer [34]. They exit the cell cycle, fuse, and give rise to a multinucleated syncytiotrophoblast. This process starts at 12 weeks of gestation and continues until term. It sustains the placental villi’s extensive growth, the overall development of the placenta, and chorionic villi repair [35]. Therefore, trophoblast senescence is a physiological phenomenon and progresses as the pregnancy advances to term. The mature placental syncytiotrophoblast presents molecular markers of cellular senescence [36,37]. They will be described in detail in the following section of this review. These markers include an increased expression of ß-galactosidase (SA-ß-gal), p16, p21, and p53; the development of heterochromatin foci; the activation of the mammalian target rapamycin complex; and telomere shortening [1].

Not only the trophoblast displays signs of aging. This process has also been described in maternal decidual cells and fetoplacental membranes. This may play an important role in the initiation of labor as the pregnancy approaches term [38,39]. The increased expression of p53, p21, senescence-associated secretory phenotype (IL-6 and IL-8), and SA-ß-gal have been found in maternal decidua and fetal membranes [39,40].

## 4. Role of Pathological Placental Aging in the Genesis of Obstetric Complications

Premature, accelerated senescence and aging can be triggered by different stressing factors, resulting in a clinical presentation of placental pathology. The clinical presentation will be a combination of the dosage and timing of the stressing agent. Brosens et al. presented a hypothesis that all “great obstetrical syndromes” result from abnormal deep placentation [41,42]. These syndromes include preeclampsia, fetal growth restriction, preterm birth, premature rupture of membranes, fetal death, placental abruption, and late abortion. All these syndromes have an early or late presentation during pregnancy. Understanding the role of placental aging could help delineate between these distinct clinical presentations. Abnormalities of deep placentation are related to an early injury of vascular endothelium caused by oxidative and endoplasmic reticulum stress. This, in return, triggers cellular senescence. The degree of initial injury can compromise at different levels the nutrient transport between the mother and the fetus. This will affect the levels of stress that the placenta is exposed to. This is an important aspect explaining the different clinical presentations of obstetric syndromes. Low levels of stress only induce adaptive responses, including the upregulation of antioxidants and call turnover by apoptosis and autophagy [34]. Moderate levels of stress may affect stem cell behavior and reduce cell proliferation. High levels and chronic stress results in the induction of proinflammatory cytokines, antiangiogenic factors, and the acceleration of trophoblast senescence [43,44].

In preeclampsia, which is probably the best-studied pregnancy pathology, several aging markers have been described. Their expression correlates with clinical presentation. Early preeclampsia and preeclampsia coexisting with fetal growth restriction have been shown to present a higher expression of senescent markers [45,46,47]. Preeclamptic pregnancies demonstrate an increased formation of nuclear aggregates (SAHF); increased DNA oxidation; and an increased expression of p53, p21, p16, and proinflammatory cytokines. Additionally, preeclampsia studies have demonstrated shorter telomeres, reduced the telomerase activity, and increased the senescence-associated secretory phenotype compared to non-preeclamptic pregnancies [45,48,49].

In preterm birth, on the other hand, accelerated placental aging may be involved in triggering the earlier onset of labor. Preterm birth may result from a spontaneous onset of contractions or premature rupture of the membranes (pPROM). Labor is associated with the expression of senescence signals in placental membranes. These include telomere length reduction; an increased expression of p53, p21, IL-6, and IL-8; and SA-ß-gal. The latter is mediated by activating the p38 mitogen-activated protein kinase (p38 MAPK) pathway. Additionally, telomeric DNA fragments released by senescent cells into the amniotic fluid may induce the p38 MAPK pathway and stimulate parturition by releasing sterile inflammatory signals. Oxidative DNA damage by ROS has been found to contribute to preterm labor and pPROM. This is probably mediated via the senescence-associated secretory phenotype [39,40,50,51].

Infection and inflammation have been described as the fundamental causes of preterm birth. Interestingly, true inflammation of the fetal membranes (chorioamnionitis) is not necessary for spontaneous preterm birth. Chorioamnionitic membranes from such preterm births have been found to show senescence signs, such as increased levels of gene encoding p21 (CDKN1A) and SA-ß-gal, as well as the downregulation of CDK and cyclins [52,53].

## 5. Causes and Biomarkers of Placental Senescence and Aging

The leading causes of premature senescence are telomere dysfunction, DNA damage, epigenomic disruption, strong mitogenic signals, reactive oxygen species (ROS), and oncogenes [20]. Telomeres are highly conserved repetitive DNA regions found at the terminal ends of chromosomes. They become progressively shorter with each mitotic cycle. Their length can also be affected by environmental factors such as smoking, hypoxia, hyperglycemia, or oxidative stress. Once they reach a critically short length, it triggers cell senescence and apoptosis. The telomerase enzyme regulates the telomere length. It is a reverse transcriptase that can add telomeric repeats to the end of the chromosome. This provides cell integrity and stability. It consists of two components, a catalytic protein component and an RNA template component. The loss of telomerase activity leads to a progressive shortening of telomeres that triggers cellular senescence when a critically short telomere length is reached. Telomere shortening, as well as telomerase activity, are important studied biomarkers of senescence [22,23,54].

Premature senescence can also be triggered by DNA damage, the DNA damage response, telomere uncapping, and telomere dysfunction. Oxidative stress triggered by ROS can induce this. ROS causes end-to-end fusion and the aggregation of telomeric DNA. Moreover, it stimulates senescence through DNA damage and activating the p53–p21 and p16-pRB signaling transduction pathways. The activation of these pathways leads to cell cycle arrest. The levels of p16, p53, and p21 have also been studied as biomarkers [39,40,51,55].

SA-β-gal is a well-described marker of detecting senescence in in vitro culture cells and tissues. Its activity can be easily detected by immunochemistry. It is specifically expressed in senescent cells and increases in an age-dependent manner in human tissues [37,56].

Senescence-associated heterochromatin foci (SAHF) is a senescence biomarker that can be visualized under microscopy in the nucleus. The p16 and p53 pathways trigger SAHF formation. They also silence the E2F target gene that regulates the progression of the cell cycle. SAHF regions are enriched by transcription silencing histones and chromatin regulators such as HIRA, Asfl, and HMGA, which all are markers of senescence [57].

Finally, an important marker of senescence is the senescence-associated secretory phenotype. It is described as a release of inflammatory signals resembling the local tissue or cellular immune response. The expression of IL-6 and IL-8 has been extensively studied as biomarkers for measuring senescence [27,58].

The markers of placental aging in different obstetric complications are summarized in Table 1.

## 6. Pathways of Placental Aging in Late Fetal Growth Restriction

To better understand the role of placental aging in late fetal growth restriction, it needs to be defined. Placental insufficiency (or uteroplacental vascular insufficiency) is a complication of pregnancy when the placenta is unable to deliver an adequate supply of nutrients and oxygen to the fetus. The Delphi consensus from 2016 has differentiated and defined early and late fetal growth restrictions [5]. According to this consensus, growth restrictions cannot be solely determined by the fetal birth weight but is a combination of estimated fetal weight and ultrasound measured Doppler parameters [5]. A critical aspect of differentiating early and late fetal growth restrictions is its coexistence with preeclampsia [59]. It complicates almost 75% of early FGR, while only less than a third of late FGR [60]. The two main late forms of smallness are FGR and small for gestational age (SGA) fetuses [61]. Late FGR, in comparison to SGA, presents Doppler abnormalities in the fetoplacental circulation but also carries a higher risk of intrauterine death [61,62,63]. On the other hand, both FGR and SGA are associated with intrauterine programming, including abnormal neurodevelopment, increased risk of metabolic and cardiovascular diseases, and including obesity [54,55,56].

Placental dysfunction is the best-known proxy for fetal smallness [41]. Interestingly 25% of pregnancies complicated by FGR do not show any abnormalities upon histopathological examination [64]. Studies have shown that placental aging in FGR pregnancies is associated with abnormal telomerase hemostasis and different cell cycle arrest patterns. Furthermore, α-klotho, an antiaging component, is decreased in pregnancies complicated by abnormal growth, preeclampsia, or both [49,65] As we have mentioned before, every placenta until term experiences some degree of aging and, consequently, presents a decreased activity that leads to normal post-term changes and promotes labor initiation. Studies of pregnancies complicated by FGR have described short telomeres, the suppression of telomerase activity, and increased apoptosis mediated by the p53 pathway [35,45,46,66,67,68] Unfortunately, many of these studies failed to differentiate between early and late FGR and FGR with or without preeclampsia, deeming these findings difficult to interpret from a clinical perspective.

Paules et al. compared markers of placental aging (telomerase activity, length, and expression of Sirtuin and Caspase genes) to show that, on a molecular level, SGA fetuses also present abnormal placental aging. This is a significant finding, because it suggests that, despite previous conclusions, SGA are constitutionally small healthy fetuses; they are also a form of fetal growth restriction. This could explain the late consequences of their abnormal growth [3].

The process of telomere length regulation by telomerase is disrupted in the placentas of SGA and FGR pregnancies. This results in reduced telomerase activity, telomere shortening, and cell senescence activation via the p53 pathway [22,69] Additionally, Paules et al. provided evidence on the role of SIRT-1 and p53 dysregulation in those placentas. SIRT1 is a stress-activated enzyme that plays a role in many nuclear pathways, such as DNA transcription, replication, and repair [3]. It acts as an antiaging agent by influencing the telomerase activity and directly inactivating the p53 pathway. The placental downregulation of SIRT-1 and p53 overexpression correlate directly with abnormal telomere hemostasis. This leads to the dysregulation of mitochondrial function, promotion of cell senescence, and apoptosis. This, in turn, is reflected in increased caspase 3 and 9 activity in FGR, which are important genes in the analysis of apoptosis [70].

The described changes are more prominent in FGR cases presenting with abnormal Doppler findings, but significant differences in the SIRT-1, p53, and telomerase activity were described in SGA fetuses. Especially, the p53 pathway is prominent in SGA, which may suggest that p53 is involved in placental aging and cell differentiation and tissue homeostasis [71]. This could explain the observed neurodevelopmental and cardiac programming changes in SGA fetuses despite the absence of histological findings confirming the presence of placental dysfunction. There has been described a linear tendency across the severity stages, supporting the hypothesis that SGA fetuses also have some degree of placental insufficiency [72,73,74,75].

## 7. Pathways of Placental Aging in Stillbirth

Stillbirth is defined as the death of a fetus weighing more than 500 g or greater than 22 weeks. WHO recommends reporting data only for fetuses stillborn above 1000 g or 28 weeks of gestation [76]. Classifications have been developed to differentiate the causes of stillbirth [6,13,77] Once all known causes such as clinically symptomatic placental insufficiency presenting as FGR, congenital defects, chromosomal abnormalities, infection, placental abruption, or infarction are excluded, the clinicians are left with “unexplained” stillbirth. It is believed that between 25–60% of stillbirths are classified as unexplained [78,79,80].

Many studies confirm placental pathology’s significance in unexplained stillbirths, but as in late SGA, it does not always present evident symptoms before occurrences and on histopathological findings [79]. Terminal villous immaturity, degenerative vascular changes, fibrin deposition, inflammation, parenchymal thrombosis, and infarction are some of the placental lesions associated with unexplained stillbirth [76]. Oxidative stress and angiogenic imbalance have also been described as potential mechanisms of stillbirth. It has been hypothesized that they all, both independently and in combination, are triggers of premature placental senescence [81].

Ferrari et al. studied the relationship between placental telomere shortening and stillbirth. They studied telomere lengths in the placentas of both term and preterm unexplained stillbirths and compared them to term births (TB) and preterm births (PTB) with and without pPROM [81]. They found that stillbirth is associated with placental telomere attrition. Interestingly they found that telomeres are shorter in preterm birth with pPROM than that without pPROM, and it mimics that in stillbirth. A similar correlation was found in studies of another senescence activator, p38 MAPK, in PTB, TB, and pPROM. Again, the p38 MAPK phenotype was found only in pPROM, suggesting a physiological response in TB and a pathological response in pPROM [55,82]. The question to answer is why some patients with a telomere attrition result in pPROM and others in stillbirth. Perhaps this could be a combination of the timing and dose of the stressing agent and the individual inability to maintain a homeostatic balance.

This study was followed by Maiti et al. who sought to determine the oxidative stress markers of placental aging in late-term and stillbirth placentas [2]. They found that in both late-term and stillbirth placentas, there is increased oxidative damage to DNA and lipids (8-hydroxy-deoxyguanosine and 4-hydroxynonenal—markers of DNA and lipid oxygenation) and an increased expression of the aldehyde oxidase enzyme responsible for oxidative damage [2]. They have also shown that the syncytiotrophoblasts of late-term placentas and stillbirths resemble the lysosomal positioning of cells under nutritional stress. This correlates with the presence of large autophagosomes and inhibition of the autophagic, cellular recycling process in the placenta. Furthermore, these autophagosomes are coated with an oxidized lipid that inhibits a fusion with lysosomes and results in abnormal protein accumulation and disrupts the syncytiotrophoblast function [83].

Stillbirths and FGR have been strongly associated with maternal smoking, which has been found to promote premature aging of the placenta. Reduced telomere lengths are found in smoking mothers’ fetuses, and similar findings are expected in the placentas [84,85].

## 8. Implications for Future Research of Late Placental Pathologies

Cellular senescence plays an important role in the development of the placenta. It can have both a positive and negative effect on the developing placenta. Depending on the timing and dosage of stress factors early, accelerated senescence can be triggered, resulting in the clinical presentation of pregnancy complications.

Most biomarkers discussed previously are drawn from studies of term or post-term placentas, which is too late to predict late FGR or stillbirths. However, cell-free DNA is currently widely used in the prenatal diagnosis [86]. It is of placental origin and can be found in maternal blood as early as 10 weeks of pregnancy. Perhaps this material could be used to assess the telomere length and predict the susceptibility to premature aging [87].

Future studies of late placental pathologies are essential to distinguish between the early and late presentations of a pregnancy complication. For fetal growth restrictions, it is the onset of symptoms before or after 32 weeks of gestation. Similarly, this age could be considered for defining early and late stillbirths. The Doppler assessment of the studied pregnancies and differentiation between SGA and FGR are also very important aspects of the study design to better understand the subtle effects of placental insufficiency that trigger early placental aging. As shown in the example, the molecular assessment of senescence markers could be the only way of diagnosing the presence of placental insufficiency. This is the only method explaining the long-term sequelae of abnormal growth.

Late or even term stillbirths rarely present Doppler or histological signs of placental insufficiency. Yet, the majority of term stillbirths occur in normally grown fetuses [6]. It has been shown that appropriate for gestational age fetuses (AGA) can also present symptoms of FGR. This was demonstrated in studies looking at the catch-up growth in fetuses with a described decreased growth velocity without other signs of placental insufficiency [88,89]. The assessment of placental senescence markers in AGA fetuses presenting a decreased growth velocity with and without stillbirth could very well shed light on the pathology of unexplained stillbirths.

The most significant bias of the presented studies was either small study groups or a lack of sufficient clinical information regarding the studied placentas. Table 2 proposes inclusion and exclusion criteria for placental aging studies of growth abnormalities and stillbirths. These are based on the current recommendations and clinical research consensus.

## 9. Conclusions

Different degrees of abnormal placentation result in the severity of fetal growth restriction and its sequelae, including fetal death. The term stillbirth of an otherwise healthy fetus is considered the most tragic pregnancy complication. The prediction and prevention of these pathologies could reduce the tremendous psychological and financial burdens on medical care systems. Understanding the role of placental aging and senescence and the development of predictive markers for these pregnancy complications could be an essential milestone for perinatal medicine care.

## Figures and Tables

**Table 1:** Summary of the obstetric complications with known roles of placental aging and their markers.

Obstetric Complication	Markers
**Preeclampsia**	Short telomeres; telomere aggregation; dysfunction; reduced telomerase activity; senescence-associated secretory phenotype; increased expression p53, CDK, p16, and p21; increased aggregation of SAHF, and increased DNA oxidation of 8-OHdG
**Preterm birth**	Short telomeres; increased expression of p53, p21, IL-6, and IL-8; and SA-ß-gal. The latter is mediated by activating the p38 mitogen-activated protein kinase (p 38 MAPK) pathway.
**Premature rupture of membranes**	Oxidative DNA damage, senescence-associated secretory phenotype, and p38 MAPK
**Chorioamnionitis**	Increased levels of gene encoding p21 (CDKN1A) and SA-ß-gal and downregulation of CDK and cyclins
**Fetal growth restriction**	Short and decreased telomerase activity, length, downregulation of SIRT1, overexpression of p53, and increased activity of Caspase 3 and 9
**Stillbirth**	Short telomeres and decreased telomere activity and increased oxidative damage to DNA and lipids

**Table 2:** Recommended definitions and inclusion/exclusion criteria for placental aging research in growth abnormalities and stillbirth.

**Fetal Growth Restriction**	
**Inclusion**	**Inclusion should be based on ultrasound criteria** SGA: EFW between 3–10 centile and normal Doppler indices (UtA, UA, and MCA) Early FGR: symptomatic < 32 weeks; EFW/AC< 3rd centile or AEDV; EFW/AC 3–10 centile with UtA PI > 95th centile, UA > 90th centile Late FGR: symptomatic > 32 weeks; EFW/AC< 3rd centile; EFW/AC 3–10 centile or reduced growth velocity (>2 quartiles) with CPR< 5 centile or UA > 95 centile Differentiate between early/late FGR/SGA with and without preeclampsia
**Exclusion**	Congenital abnormalities, chromosomal abnormalities, TORCH infection, spontaneous preterm birth, premature rupture of membranes, other maternal infections, early neonatal sepsis, placental abruption, and diabetes
**Stillbirth**	
**Inclusion**	Unexplained stillbirth Differentiate between below/above 32 weeks and if with known ultrasound criteria of FGR (as above) Classify by birthweight: SGA weight < 10th centile, AGA weight 10th -90th centile, LGA > 90th centile
**Exclusion**	Congenital abnormalities, chromosomal abnormalities, TORCH infection, spontaneous preterm birth, premature rupture of membranes, other maternal infections, fetal sepsis, intrapartum death, early neonatal death, any other known causes of fetal death, preeclampsia, diabetes, and placental abruption

EFW: estimated fetal weight; AC: abdominal circumference; AEDV: absent end diastolic velocity; UtA: uterine artery; PI: pulsatility index; UA: umbilical artery; TORCH: toxoplasmosis, rubella, cytomegalovirus, herpes; SGA: small for gestational age; AGA: appropriate for gestational age; LGA: large for gestational age; CPR: cerebroplacental ration; and MCA: middle cerebral artery.
